# Taxonomic and Functional Fecal Microbiota Signatures Associated With Insulin Resistance in Non-Diabetic Subjects With Overweight/Obesity Within the Frame of the PREDIMED-Plus Study

**DOI:** 10.3389/fendo.2022.804455

**Published:** 2022-04-28

**Authors:** Alessandro Atzeni, Thomaz F. S. Bastiaanssen, John F. Cryan, Francisco J. Tinahones, Jesús Vioque, Dolores Corella, Montserrat Fitó, Josep Vidal, Isabel Moreno-Indias, Ana M. Gómez-Pérez, Laura Torres-Collado, Oscar Coltell, Olga Castañer, Monica Bulló, Jordi Salas-Salvadó

**Affiliations:** ^1^ Department of Biochemistry and Biotechnology, Universitat Rovira i Virgili, Reus, Spain; ^2^ Institut D’Investigació Sanitària Pere Virgili (IISPV), Hospital Universitari de Sant Joan de Reus, Reus, Spain; ^3^ Centro de Investigación Biomédica en Red de Fisiopatología de la Obesidad y la Nutrición (CIBEROBN), Instituto de Salud Carlos III, Madrid, Spain; ^4^ APC Microbiome Ireland, Department of Anatomy and Neuroscience, University College Cork, Cork, Ireland; ^5^ Unidad de Gestión Clínica de Endocrinología y Nutrición, Instituto de Investigación Biomédica de Málaga (IBIMA), Hospital Universitario Virgen de la Victoria, Málaga, Spain; ^6^ Instituto de Investigación Sanitaria y Biomédica de Alicante, ISABIAL-Universidad Miguel Hernández (UMH), Alicante, Spain; ^7^ Centro de Investigación Biomédica en Red Epidemiología y Salud Pública (CIBERESP), Instituto de Salud Carlos III (ISCIII), Madrid, Spain; ^8^ Department of Preventive Medicine, University of Valencia, Valencia, Spain; ^9^ Cardiovascular Risk and Nutrition (Regicor Study Group), Hospital del Mar Research Institute (IMIM), Barcelona, Spain; ^10^ Endocrinology and Nutrition Department, Institut d’Investigacions Biomèdiques August Pi Sunyer (IDIBAPS), Hospital Clinic Universitari, Barcelona, Spain; ^11^ Centro de Investigación Biomédica en Red Diabetes y Enfermedades Metabólicas (CIBERDEM), Instituto de Salud Carlos III (ISCIII), Madrid, Spain; ^12^ Department of Computer Languages and Systems, University Jaume I, Castelló de la Plana, Spain

**Keywords:** fecal microbiota, insulin resisitance, HOMA-IR, 16S sequencing, gut metabolic modules

## Abstract

**Objective:**

An altered gut microbiota has been associated with insulin resistance, a metabolic dysfunction consisting of cellular insulin signaling impairment. The aim of the present study is to determine the taxonomic and functional fecal microbiota signatures associated with HOMA-IR index in a population with high cardiovascular risk.

**Methods:**

A total of 279 non-diabetic individuals (55–75 years aged) with overweight/obesity and metabolic syndrome were stratified according to tertiles of HOMA-IR index. Blood biochemical parameters, anthropometric measurements and fecal samples were collected at baseline. Fecal microbial DNA extraction, 16S amplicon sequencing and bioinformatics analysis were performed.

**Results:**

*Desulfovibrio*, *Odoribacter* and *Oscillospiraceae* UCG-002 were negatively associated with HOMA-IR index, whereas predicted total functional abundances revealed gut metabolic modules mainly linked to amino acid degradation. *Butyricicoccus*, *Erysipelotrichaceae* UCG-003, *Faecalibacterium* were positively associated with HOMA-IR index, whereas predicted total functional abundances revealed gut metabolic modules mainly linked to saccharide degradation. These bacteria contribute differentially to the gut metabolic modules, being the degree of contribution dependent on insulin resistance. Both taxa and gut metabolic modules negatively associated to HOMA-IR index were linked to mechanisms involving sulfate reducing bacteria, improvement of intestinal gluconeogenesis and production of acetate. Furthermore, both taxa and gut metabolic modules positively associated to HOMA-IR index were linked to production and mechanisms of action of butyrate.

**Conclusions:**

Specific taxonomic and functional fecal microbiota signatures associated with insulin resistance were identified in a non-diabetic population with overweight/obesity at high cardiovascular risk. These findings suggest that tailoring therapies based on specific fecal microbiota profiles could be a potential strategy to improve insulin sensitivity.

## Introduction

Insulin is a peptide hormone that plays an essential role in the process of glucose metabolism regulation. Insulin resistance (IR) can be defined as a pathological condition of cellular insulin signaling impairment with consequent disturbance of intracellular signaling transduction, which affects several organs and tissues (1, 2). Visceral adiposity and increased body fat induce the release of relatively high levels of free fatty acids and pro-inflammatory cytokines into circulation, causing the development of hepatic and muscle IR and type 2 diabetes (T2D) (3).

An altered gut microbiota composition has been associated with the pathophysiology of obesity and IR (4). First evidence was reported in 2004 by Bäckhed et al., who has observed an increase in body fat and IR in germ-free mice transplanted with gut microbiota from conventionally raised mice donors (5). A few years later, Vrieze et al., shown that insulin sensitivity improved in a cohort of human participants with metabolic syndrome (MetS) six weeks after receiving microbiota from lean healthy human donors (6).

The gut microbiome can induce IR through different mechanisms. Cani et al., were the first to identify bacterial lipopolysaccharide (LPS) as a causative inflammatory factor of IR (7). LPS increases intestinal permeability with its consequent translocation from the intestinal lumen to circulation (8, 9). At the cell membrane level, LPS binds and activates the toll-like receptor 4 (TLR4), with consequent downstream cell signaling pathway activation which lead to inflammatory response and cytokine expression and secretion (10). A reduction of bacterial species able to produce short chain fatty acids (SCFAs), with well-established anti-inflammatory effects (11–13), also induces IR (4). However, these findings are still not totally clear due to some controversial results, as those described by Perry and colleagues, who observed increased production of acetate associated with IR and obesity in rodents exposed to high caloric diet (14). In addition, the bioconversion of bile acids carried out by bacteria from human gut, including members of *Lactobacilli*, *Bifidobacteria*, *Clostridium* and *Bacteroides*, has been shown contributing to glucose homeostasis (15, 16). A study conducted by Pedersen et al., aiming to identify the gut microbial profile of non-diabetic individuals with IR, demonstrated a positive association between IR and branched-chain amino acids (BCAA) levels (15). Taken together, these studies suggest that gut microbiota may play an important role in modulating IR, though it remains unclear which aspects of the microbiota contribute to this. Therefore, the identification of potential fecal microbiota profiles and related metabolic pathways linked to the development of IR is crucial, as the characterization of the intestinal bacterial community related to glucose homeostasis would be a useful strategy to ameliorate IR and related disorders (17). Accordingly, the aim of the present study was to determine the taxonomic and functional fecal microbiota signatures associated with IR in a population of 279 non-diabetic elderly individuals with overweight/obesity and MetS.

## Patients and Methods

### Participants and Study Design

This cross-sectional study was conducted with baseline data of participants recruited for the PREDIMED-Plus study (18, 19), an ongoing parallel-group, randomized and controlled clinical trial conducted in 23 Spanish centers, which aims to evaluate the effect of an intensive weight loss intervention, based on an hypocaloric Mediterranean diet (MedDiet), physical activity promotion and behavioral support, on cardiovascular disease (CVD) events, compared to a control group receiving usual care advice. The trial was registered at the International Standard Randomized Controlled Trial (http://www.isrctn.com/ISRCTN89898870) and approved by the institutional review board of all participating institutions. The full detailed PREDIMED-Plus protocol is available at http://www.predimedplus.com.

PREDIMED-Plus eligible participants were men and women aged 55–75 years, with overweight or obesity (body mass index (BMI) 27–40 kg/m^2^), who satisfied at least three criteria for the MetS (waist circumference > 102 cm in men and > 88 cm in women; serum triglyceride ≥ 150 mg/dL or drug treatment for elevated triglycerides; high-density lipoprotein (HDL) cholesterol < 40 mg/dL in men and < 50 mg/dL in women or drug use for low HDL cholesterol; blood pressure ≥ 130/85 mmHg or antihypertensive drug treatment; and fasting plasma glucose level ≥ 100 mg/dL or hypoglycemic treatment) and were free of CVD.

A subsample of 400 participants from the PREDIMED-Plus recruiting centers of Reus and Malaga (Spain) were randomized and matched for age, sex and BMI. For the present cross-sectional study, we selected 279 individuals with available stool samples at baseline and without T2D.

### Clinical Assessments and Blood Biochemical Analysis

Body weight was measured using high-quality electronic calibrated scales, height was measured using a wall-mounted stadiometer, waist circumference was measured midway between the lowest rib and the iliac crest using an anthropometric tape, blood pressure was measured using a validated semiautomatic oscillometer (Omron HEM-705CP, Kyoto, Japan).

Blood samples were collected after overnight fasting. Plasma levels of glucose, insulin, total cholesterol, HDL cholesterol and triglycerides were measured using standard enzymatic methods, low-density lipoprotein (LDL) cholesterol was calculated with the Friedewald formula (whenever triglycerides were less than 300 mg/dL), glycated hemoglobin was measured by a chromatographic method. Insulin resistance was estimated by the homeostatic model assessment of insulin resistance (HOMA-IR index) (20), using the following formula:


insulin(mU/mL)×glucose(mg/dL)405

In addition, information regarding lifestyle, education level, disease prevalence and current medication use was collected, whereas a validated 17-item questionnaire (21) was used to assess the adherence to the MedDiet.

### Fecal DNA Extraction and 16S rRNA Gene Sequencing

**Figure 1:**
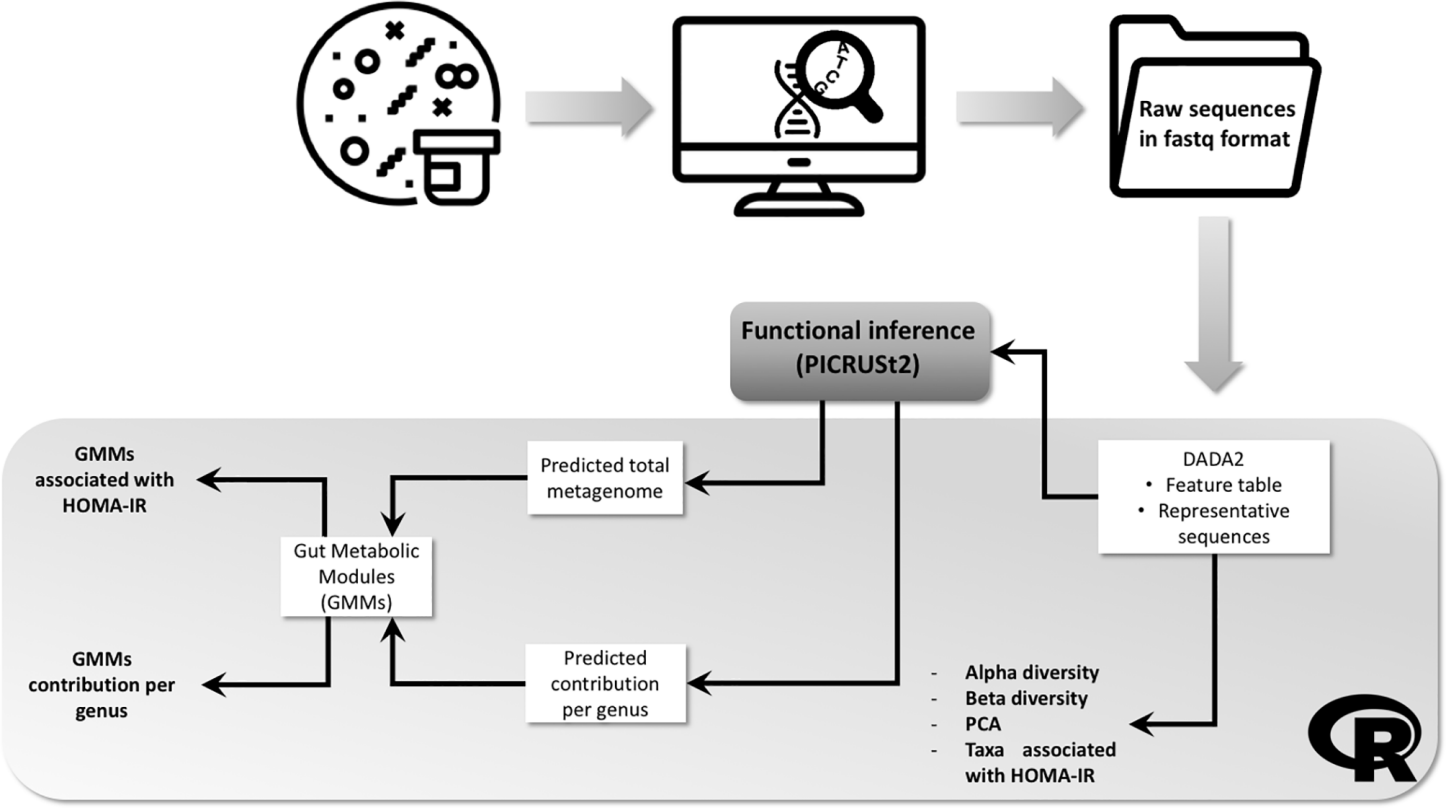
Workflow showing the fecal samples analytic process and bioinformatics pipeline. Bacterial DNA was extracted from frozen fecal and 16S amplicon sequencing performed. Resulting raw sequences in fastq format were imported into R environment and processed with DADA2 package. Output files followed 3 different pipelines: (1) processed in order to obtain information about alpha and beta diversity and differential abundant taxa; (2) processed with PICRUSt2 in order to obtain the predicted total functional abundances, then gut metabolic modules (GMM) were computed and the association with HOMA-IR index determined; (3) processed with PICRUSt2 in order to obtain the predicted contribution per genus of each GMM previously computed.

A complete workflow including the analytic steps is shown in **Figure 1**.

Stool samples were collected at home by participants and kept frozen. In case of antibiotic use or prebiotics supplementation, samples were collected 15 days after completion of treatment. A time range of 15 days was considered wide enough to gut microbiota recovery after therapeutic antibiotic usage, taking into account the exposure to short-term treatment with amoxicillin (22). Once delivered to the laboratory, stool samples were separated into 250 mg aliquots and stored at −80°C until analysis.

Microbial DNA was extracted with the QIAmp PowerFecal DNA kit (Qiagen, Hilden, Germany), following the manufacturer’s instructions, and DNA concentration and purity assessed with the Qubit 2.0 Fluorometer dsDNA Broad Range Assay Kit (Thermo Fisher Scientific, Waltham, MA, USA). Targeted 16S sequencing libraries were created with the 16S Metagenomics kit (Thermo Fisher Scientific, Waltham, MA, USA), using a pool of primers to amplify the regions V2–V9 of the 16S rRNA gene, and barcoded adapters ligated using the Ion Plus Fragment Library Kit (Thermo Fisher Scientific, Waltham, MA, USA). Synthesized libraries were pooled and templated on the automated Ion Chef system (Thermo Fisher Scientific, Waltham, MA, USA) and sequenced on the Ion S5 (Thermo Fisher Scientific, Waltham, MA, USA). Reads generated were subsequently converted in fastq format using the File Explorer plugin of the Torrent Suite Server software (Thermo Fisher Scientific, Waltham, MA, USA), interfaced with the Ion S5. The quality control of fastq files was assessed with FastQC using a quality score of 20 as a threshold (https://www.bioinformatics.babraham.ac.uk/projects/fastqc/).

### Statistical Analysis

Demultiplexed sequence reads, were imported into R (version 4.0.5), quality filtered, chimera-checked and clustered in amplicon sequence variants (ASVs) using the R package DADA2 (v 1.16.0) (23) whereas taxonomy was assigned using the SILVA reference database release 138 (24). Obtained counts were filtered and transformed using a centered log-ratio (CLR) transformation (25). Principal component analysis (PCA) was performed to evaluate the distribution of the study population. Alpha diversity indices, Chao1 (26), Shannon (27) and Simpson (28) were calculated and differences between tertiles of HOMA-IR index tested. Beta diversity was computed in terms of Aitchison distance, or Euclidean distance over CLR-transformed counts (25). Permutational multivariate analysis of variance (PERMANOVA) was performed using the adonis function (“vegan” package, version 2.5-6) to test differences between groups composition. The association between taxa and tertiles of HOMA-IR index was tested using generalized linear modelling (GLM), adjusted for 17-item MedDiet adherence score and recruiting center as covariates. Multiple testing correction was performed using Storey’s q-value procedure, and *q* < 0.1 deemed as significant (29).

In order to perform an inferential analysis of the functional potential of the microbiome, DADA2 output files were parsed and used as input for PICRUSt2 in order to generate a table of inferred per-sample abundances of KEGG genome orthologs (KO) (30). To assess the diversity in metabolic potential and anaerobic fermentation capacity encoded in (meta-) genomic sequences, we used a manually curated database of human gut metabolic modules (GMM), that describe enzymatic processes annotated using exclusively prokaryotic and archaeal KO (31). GMM counts were filtered, converted to CLR and the association with HOMA-IR index tested with the same GLM setup used for the analysis of taxa.

In addition to the predicted total functional abundances, PICRUSt2 also includes the predicted functional contribution per taxa (using the –stratified flag). This information was used to study the contribution of each taxon of interest to the GMM of interest.

The clinical characteristics of the study population were described using IBM SPSS Statistics version 23 (SPSS Inc., Chicago, Illinois, USA). Data normality was assessed with Shapiro-Wilk test. Normally distributed variables were described by means and standard deviations, non-normally distribute variables by median and interquartile range, whereas categorical variables were described as number and percentages. Differences across tertiles were evaluated through one-way analysis of variance (ANOVA) parametric test or Kruskal-Wallis non-parametric test for numerical variables, and with Pearson’s chi-square test for categorical variables. Student’s t parametric test or Mann-Whitney non-parametric test was used to calculate differences between tertiles for numerical variables. All statistical tests were 2-sided and *p* < 0.05 was deemed statistically significant.

## Results

### Clinical Characteristics of the Study Population

Clinical characteristics of the study population categorized by tertiles of HOMA-IR index are shown in **Table 1**. Body weight and waist circumference were significantly lower in those participants in tertile 1 (T1) of HOMA-IR index than those in tertile 2 (T2) or tertile 3 (T3); BMI, triglycerides, glucose, insulin and glycated hemoglobin were significantly lower in those participants in T1 compared to T2 and T3 and participants in T2 *versus* those in T3; HDL-cholesterol was significantly higher in T1 *versus* T2 and T3, and in T2 *versus* T3; MedDiet adherence score was significantly higher in T2 vs T3.

**Table 1:** Characteristics of the study population according to tertiles of HOMA-IR index.

	Tertile 1 (n = 93)	Tertile 2 (n = 93)	Tertile 3 (n = 93)	*p* trend^&^
Female sex	51 (54.8)	53 (57.0)	47 (50.5)	0.668
Age (years)	66.0 [7.0]	65.0 [7.0]	65.0 [8]	0.619
Recruiting center				0.817
Málaga	33 (35.5)	36 (38.7)	32 (34.4)	
Reus	60 (64.5)	57 (61.3)	61 (65.6)	
Body weight (kg)	82.9 [12.6]	87.6 [18.3]*	90.0 [16.4]**	< 0.001
BMI (kg/m^2^)	30.7 [4.6]	33.3 [4.8]**	34.3 [6.0]**†	< 0.001
Waist circumference (cm)	105.9 ± 8.2	109.0 ± 10.3*	111.7 ± 9.8**	< 0.001
SBP (mmHg)	139.8 [22.1]	138.0 [19.0]	140.7 [31.3]	0.852
DBP (mmHg)	79.8 ± 10.3	79.8 ± 8.3	79.1 ± 11.1	0.868
Total cholesterol (mg/dL)	209.0 [48.0]	204.0 [51.0]	200.0 [42.0]	0.641
HDL-cholesterol (mg/dL)	54.0 [17.0]	49.0 [11.0]*	46.0 [11.0]**†	< 0.001
LDL-cholesterol (mg/dL)	124.0 [42.0]	117.0 [44.0]	116.0 [38.0]	0.211
Triglycerides (mg/dl)	118.5 [69.0]	153.0 [83.0]**	175.0 [107.0]**	< 0.001
FPG (mg/dL)	93.0 [15.0]	100.0 [17.0]**	108.0 [20.0]**†	< 0.001
Fasting insulin (mU/mL)	10.8 [3.4]	16.6 [3.0]**	27.6 [10.5]**††	< 0.001
Glycated hemoglobin (%)	5.6 [0.4]	5.7 [0.5]*	5.8 [0.6]**†	< 0.001
HOMA-IR index	2.4 [0.7]	4.1 [1.0]**	7.3 [2.7]**††	< 0.001
MedDiet adherence score	8.0 [3.0]	8.0 [2.0]	7.0 [3.0]†	0.009
Hypercholesterolemia	55 (59.1)	63 (67.7)	56 (60.2)	0.419
Hypertension	87 (93.5)	86 (92.5)	88 (94.6)	0.837
Smoking habits				0.282
Current smoker	12 (12.9)	13(14.0)	15 (16.1)	
Former smoker	29 (31.2)	30 (32.3)	40 (43.0)	
Never smoker	52 (55.9)	50 (53.8)	38 (40.9)	
Education				0.641
Primary school	50 (53.8)	56 (60.2)	46 (49.5)	
Secondary school	30 (32.3)	28 (30.1)	34 (36.6)	
University	13 (14.0)	9 (9.7)	13 (14.0)	

HOMA-IR, homeostatic model assessment for insulin resistance; BMI, body mass index; SBP, systolic blood pressure; DBP, diastolic blood pressure, HDL, high-density lipoprotein; LDL, low-density lipoprotein; FPG, fasting plasma glucose; MedDiet, Mediterranean diet. Data presented as mean ± SD, median [IQR] or number (percentage). ^&^One-way ANOVA, Pearson’s chi-square test or Kruskal-Wallis test was used to calculate differences across tertiles, Student’s t-test or Mann-Whitney test was used to calculate differences between tertiles. ** p < 0.001 vs tertile 1; * p < 0.05 vs tertile 1; †† p < 0.001 versus tertile 2; † p < 0.05 versus tertile 2.

### Microbiome Diversity Is Not Associated With HOMA-IR Index

PCA analysis over the CLR-transformed microbiome count data revealed that the first two components account approximately 12.22% and 8.46% respectively. These figures are approximately in line with literature (32). PCA plots shown that the microbiome sample distribution did not cluster based on tertiles of HOMA-IR index (**Supplementary Figure 1**). There were no statistically significant differences across tertiles of HOMA-IR index in the alpha diversity indices Chao1, Shannon and Simpson (**Supplementary Figure 2** and **Supplementary Table 1**). The results of the PERMANOVA analysis and the permutation test for homogeneity of multivariate dispersions using Aitchison distance were also not statistically significant across tertiles of HOMA-IR index (**Supplementary Table 2**).

### Several Microbial Genera and GMM Are Associated With HOMA-IR Index

Taxa and GMM significantly associated with HOMA-IR index are summarized in **Table 2**. Genera *Desulfovibrio*, *Odoribacter* and *Oscillospiraceae* UCG-002 were negatively associated with the highest tertile of HOMA-IR index, whereas genera *Butyricicoccus*, *Erysipelotrichaceae* UCG-003 and *Faecalibacterium* were positively associated with the highest tertile of HOMA-IR index. In addition, 21 GMM generated from the predicted total functional abundances were associated with the highest tertile of HOMA-IR index, 8 of these GMM were negatively associated with the highest tertile of HOMA-IR index, whereas 14 GMM were positively associated.

**Table 2:** Taxa and GMM associated with tertile 3 of HOMA-IR index.

Taxa	Effect size	*q-*value	Association with T3
Desulfovibrio	−0.724	0.023	Negative
Odoribacter	−0.832	0.043	Negative
Oscillospiraceae UCG-002	−0.806	0.088	Negative
Erysipelotrichaceae UCG-003	0.487	0.088	Positive
Faecalibacterium	0.530	0.088	Positive
Butyricicoccus	0.605	0.088	Positive
**GMM**			
Alanine degradation I	−0.201	0.053	Negative
Pectine degradation II	−0.101	0.053	Negative
Acetate to acetyl CoA	−0.080	0.053	Negative
Aspartate degradation I	−0.071	0.053	Negative
Glycine degradation	−0.063	0.069	Negative
Cysteine degradation II	−0.061	0.070	Negative
Glutamine degradation I	−0.145	0.072	Negative
Isoleucine degradation	−0.069	0.094	Negative
Fructan degradation	0.132	0.047	Positive
Galactose degradation	0.040	0.053	Positive
Sucrose degradation I	0.049	0.053	Positive
Pyruvate formate lyase	0.049	0.053	Positive
Melibiose degradation	0.054	0.053	Positive
Threonine degradation II	0.056	0.053	Positive
Ethanol production I	0.085	0.053	Positive
Lactate production	0.094	0.053	Positive
Fructose degradation	0.111	0.053	Positive
Threonine degradation I	0.044	0.056	Positive
Lactaldehyde degradation	0.069	0.070	Positive
Bifidobacterium shunt	0.025	0.072	Positive
Glutamine degradation II	0.028	0.096	Positive
Sucrose degradation II	0.038	0.098	Positive

GLM was used to calculate the association between taxa and tertile 3 (T3) of HOMA-IR index and between predicted total functional abundances GMM and T3 of HOMA-IR index. Adjusted Storey’s q-values q < 0.1 deemed as significant.

### Altered Functions Are Regulated by Different Microbes Based on HOMA-IR Status

The contribution of each taxon to the GMM of interest has been displayed at family level (at genus level in case of taxa of interest) per sample (**Supplementary Figures 3**, **4**) and per tertile of HOMA-IR index (**Supplementary Figures 5**, **6**). For the generation of stacked barplots, just the main contributors are represented with a cutoff of 0.3, it means that if a bacterium never contributes more than 30% to any function is not considered for the plot and is categorized as “other taxa”. However, the “other taxa” were not excluded from the statistical analysis.

Just those taxa significantly contributing (*q* < 0.1) to those GMM associated with the highest tertile of HOMA-IR index in from the predicted total functional abundances were depicted.

Taxa contribute differentially to the GMM of interest depending on IR: in case of negative association with HOMA-IR index, *Desulfovibrio*, *Odoribacter*, *Oscillospiraceae* UCG-002, significantly contributes to aspartate degradation and glycine degradation (**Table 3**); in case of positive association with HOMA-IR index, *Butyricicoccus*, *Erysipelotrichaceae* UCG-003, *Faecalibacterium*, significantly contribute to glutamine degradation II, lactaldehyde degradation, lactate production and fructan degradation (**Table 4**).

**Table 3:** Taxa contribution per tertile to the GMM negatively associated with HOMA-IR index.

GMM	Taxa	T1	T2	T3	Effect size	*q-*value
Aspartate degradation I	Marinifilaceae_Odoribacter	0.0038	0.0031	0.0023	−0.6466	0.0209
	Erysipelatoclostridiaceae	0.0049	0.0067	0.0099	0.7242	0.0251
	Oscillospirales_Oscillospiraceae UCG-002	0.0535	0.0550	0.0413	−0.7908	0.0251
	Peptostreptococcaceae	0.0021	0.0039	0.0052	0.6368	0.0818
	Butyricicoccaceae	0.0017	0.0021	0.0030	0.4768	0.0872
	Desulfovibrionaceae_Desulfovibrio	0.0068	0.0054	0.0043	−0.6658	0.0872
Glycine degradation	Marinifilaceae_Odoribacter	0.0042	0.0034	0.0023	−0.5255	0.0605

T1, tertile 1 of HOMA-IR index; T2, tertile 2 of HOMA-IR index; T3, tertile 3 of HOMA-IR index. The contribution was described at family level (at genus level in case of taxa of interest) with established cutoffs of 0.3. GLM was used to calculate differences between tertiles of HOMA-IR and adjusted Storey’s q-values q < 0.1 deemed as significant.

**Table 4:** Taxa contribution per tertile to the GMM positively associated with HOMA-IR index.

GMM	Taxa	T1	T2	T3	Effect size	*q-*value
Fructan degradation	Butyricicoccaceae_Butyricicoccus	0.0058	0.0072	0.0067	0.5303	0.0993
	Bacteroidaceae	0.0288	0.0303	0.0438	−0.5268	0.0993
	Erysipelatoclostridiaceae	0.0333	0.0330	0.0290	−0.8646	0.0993
Fructose degradation	Anaerovoracaceae	0.0003	0.0002	0.0005	0.4756	0.0112
	Erysipelatoclostridiaceae	0.0146	0.0131	0.0138	−0.8037	0.0516
	UCG-010	0.0063	0.0047	0.0022	−0.6337	0.0600
Glutamine degradation II	Oscillospirales_Oscillospiraceae UCG-002	0.0518	0.0492	0.0368	−0.4279	0.0205
	Butyricicoccaceae_Butyricicoccus	0.0006	0.0008	0.0009	0.6283	0.0304
	Marinifilaceae	0.0050	0.0040	0.0033	−0.6122	0.0304
	UCG-010	0.0025	0.0017	0.0008	−0.6740	0.0367
	Erysipelatoclostridiaceae_Erysipelotrichaceae UCG-003	0.0052	0.0078	0.0093	0.7995	0.0611
	Ruminococcaceae_Faecalibacterium	0.0065	0.0095	0.0095	0.5042	0.0638
	Erysipelatoclostridiaceae	0.0079	0.0066	0.0074	−0.7875	0.0638
	Muribaculaceae	0.0062	0.0034	0.0060	−0.7419	0.0638
	Peptostreptococcaceae	0.0004	0.0007	0.0007	0.4752	0.0638
Lactaldehyde degradation	Tannerellaceae	0.0008	0.0003	0.0002	−0.4656	0.0235
	Erysipelatoclostridiaceae_Erysipelotrichaceae UCG-003	0.0073	0.0112	0.0130	0.6334	0.0619
	Erysipelatoclostridiaceae	0.0122	0.0124	0.0104	−0.7217	0.0619
Lactate production	Butyricicoccaceae_Butyricicoccus	0.0027	0.0035	0.0038	0.6145	0.0267
	Oscillospirales_Oscillospiraceae UCG-002	0.1743	0.1601	0.1281	−0.4463	0.0267
	Erysipelatoclostridiaceae	0.0208	0.0192	0.0182	−0.7919	0.0391
	UCG-010	0.0058	0.0048	0.0020	−0.6081	0.0391
Threonine degradation II	Marinifilaceae	0.0177	0.0154	0.0134	−0.6546	0.0166
	Selenomonadaceae	0.0023	0.0082	0.0122	0.6122	0.0678

T1, tertile 1 of HOMA-IRs; T2, tertile 2 of HOMA-IR index; T3, tertile 3 of HOMA-IR index. The contribution was described at family level (at genus level in case of taxa of interest) with established cutoffs of 0.3. GLM was used to calculate differences between tertiles of HOMA-IR index and adjusted Storey’s q-values q < 0.1 deemed as significant.

## Discussion

**Figure 2:**
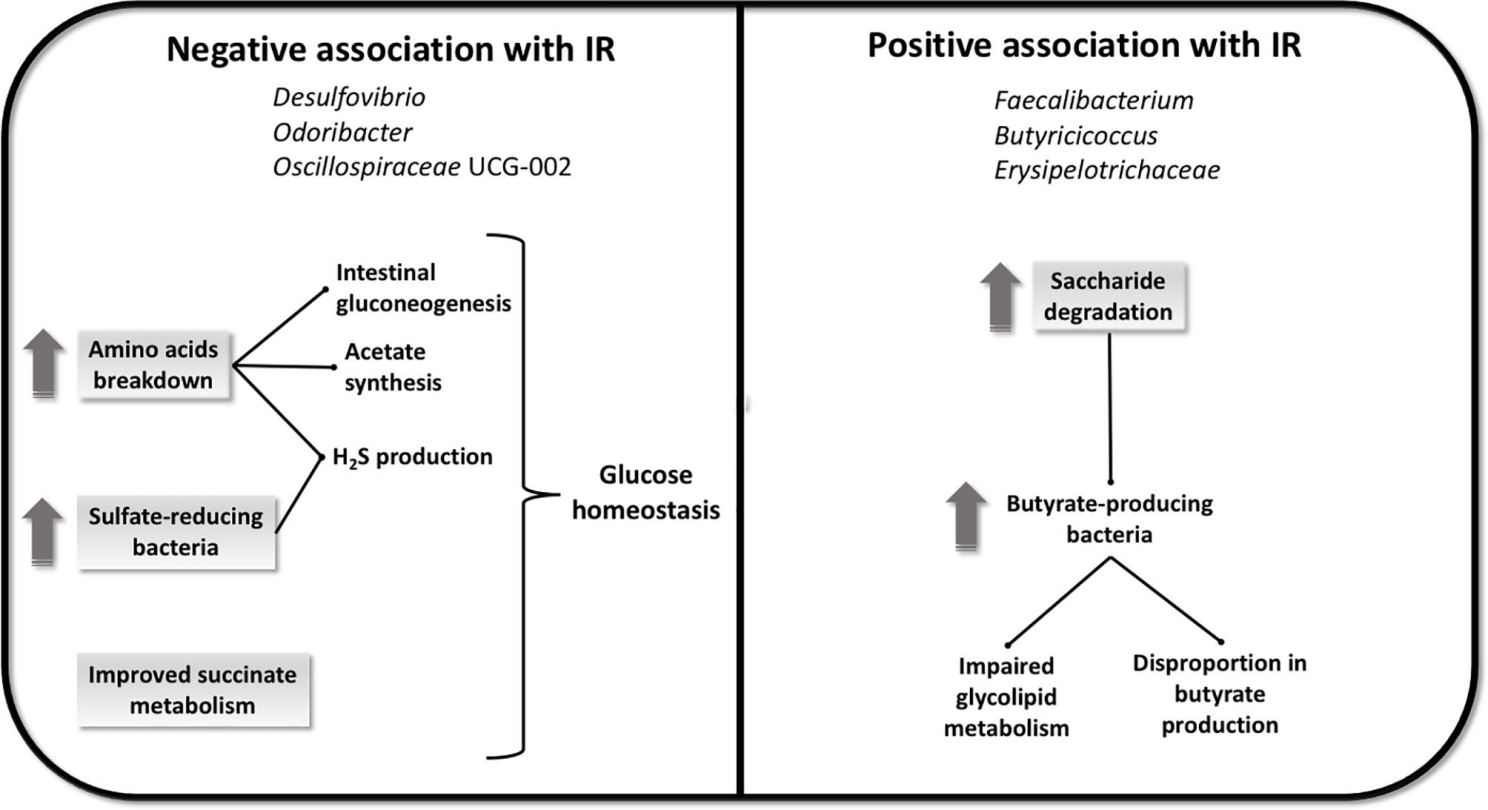
Potential mechanisms explaining the association between fecal microbiome and IR. The negative association with IR seems to be linked with glucose homeostasis, induced by an increase in amino acids breakdown and by an increase in sulfate-reducing bacteria, with consequent promotion of intestinal gluconeogenesis, acetate synthesis and H_2_S production, in addition to an improved succinate metabolism. The positive association with IR seems to be linked to an increase in saccharides degradation that can induce the growth of butyrate-producing bacteria and bring to a disproportion in butyrate synthesis and an impairment in glycolipid metabolism.

In the current study, we identified 6 different taxa consistently associated with IR. In addition, exploring the total predicted metagenome we found 21 GMM associated with IR. Finally, we explored the predicted taxa contribution to the GMM of interest, showing that bacteria contribute differentially to the function and that the degree of contribution is also dependent on IR. Therefore, we identified taxonomic signatures and potential metabolic pathways related to IR in a non-diabetic population at high cardiovascular risk. The relatively large number of altered GMM found may be understood in light of the altered gut metabolic environment one would expect to typically accompany a higher IR, thus drastically altering the potential substrates for the microbiome. Potential mechanisms explaining the association between fecal microbiota and IR are resumed in **Figure 2**. Microbial genera *Desulfovibrio*, *Odoribacter* and *Oscillospiraceae* UCG-002, were found negatively associated with IR. Notably, *Desulfovibrio*, a genus that is typically associated with worsened host health (33), was negatively associated with IR. Bacteria of the genus *Desulfovibrio* are the most represented sulfate-reducing bacteria (SRB) residing in the human gut. The excessive presence of SRB in the gut, has been associated with the development of disorders, such as inflammatory bowel disease (34) and ulcerative colitis (33). Also, 16S pyrosequencing results shown that members of *Desulfovibrionaceae* family were found significantly abundant in murine models of MetS and in animals with diet-induced obesity (35). In contrast, a significant increase of *Desulfovibrio* was described in the fecal microbiota of healthy subjects compared to individuals with obesity analysed with 16S rRNA sequencing (36), and reduced *Desulfovibrio* was also found in the gut microbiota assessed with quantitative PCR of preschool children with obesity compared to healthy controls, echoing our findings (37). In a study conducted to assess the nutritional effects as well as the adherence to the MedDiet on the gut microbiota of healthy adults analyzed with 16S rRNA gene sequencing, *Desulfovibrio* was found more abundant in lean individuals (38). Accordingly, we found *Desulfovibrio* negatively associated with the highest tertile of HOMA-IR index, who includes those subjects with a significantly higher BMI. Hydrogen sulfide (H_2_S) is a gas metabolite produced by SRB in the gut that directly activates the secretion of glucagon-like peptide-1 (GLP-1), a peptide hormone involved in glucose homeostasis and appetite regulation. Mice treated with a specific SRB supplemented prebiotic diet has elevated levels of *Desulfovibrio piger*, detected with targeted real-time PCR, and increased concentrations of H_2_S in feces and colon, with consequent stimulation of GLP-1 and enhancement of insulin secretion, improved oral glucose tolerance, and reduced food intake (39). Mice treated with liraglutide, an injectable GLP-1 receptor agonist (GLP-1 RA) used in the treatment of T2D, with demonstrated efficacy in improving glycemic control, has been shown to produce substantial changes in the gut microbiota assessed with pyrosequencing of 16S rRNA with enrichment of several genera, of whom *Desulfovibrio* (40). These findings support the potential involvement of *Desulfovibrio* in glucose homeostasis through the production of H_2_S and activation of GLP-1 and can partially explain the negative association between this genus and IR observed in our study.

In the present study HOMA-IR index was negatively associated with GMM linked to amino acid breakdown. The fraction of amino acids that are not absorbed by the colonic mucosa are metabolized by the large intestinal microbiota, resulting in the generation of metabolites and end-products such as SCFAs, and hydrogen sulfate ion (HO_4_S^−^) (41). Accordingly, we observed that HOMA-IR index was negatively associated to functions linked to cysteine degradation, resulting in the production of H_2_S. Glycine and glutamine are intestinal gluconeogenesis substrates, and it has been observed that intestinal gluconeogenesis protects against diabetes and obesity by suppressing hepatic gluconeogenesis and positively regulating glucose homeostasis (42). Furthermore, glutamine immunomodulatory effects have been described (43). In addition, we observed that in case of functions linked to aspartate degradation I and glycine degradation II, taxa of interest contribute significantly to the negative association with HOMA-IR index. Aspartate and glycine are precursors for the synthesis of acetate (44), who has been demonstrated to play a regulatory role in body weight control, and insulin sensitivity through effects on lipid metabolism and glucose homeostasis (45).

*Odoribacter* is an anaerobic nonmotile Gram-negative, succinate-consuming and SCFA-producing rod-shaped bacteria. This genus has been linked to a decrease in clinical parameters associated with CVD risk (46). The results of a cross-sectional study conducted in a cohort of Spanish subjects shown that obesity was associated with elevated levels of circulating succinate concomitant with impaired glucose metabolism. This increase was associated with specific changes in gut microbiota, assessed with 16S sequencing, including lower relative abundance of succinate-consuming *Odoribacteraceae* family in obese individuals (47). Odoribacter spp. were found to be associated with a healthy fasting serum lipid profile in women with obesity (48). Wang et al. shown that a high-fat diet induces reduction in the relative abundance of *Odoribacter* in mice fecal microbiota analysed with 16S rRNA gene sequencing, then suppressed by the use of capsaicin, suggesting that changes in the abundance of *Odoribacter* may play an important role in the effects of treatments for glucose intolerance and obesity (49). Our findings are in agreement with the beneficial characteristics described for this genus in relation to IR, as we observed a negative association with high HOMA-IR index in our study population.

*Oscillospiraceae* family negatively correlates with BMI and anti-insulin autoantibodies in type 1 diabetes in children (50). In addition, members of the family *Oscillospiraceae* were found depleted in obese children (51). Accordingly, we find UCG-002 from *Oscillospiaraceae* family negatively associated with the highest tertile of HOMA-IR index, that includes those subjects with significantly higher BMI, and other variables related to obesity and IR.

Therefore, our results suggest that genera negatively associated with IR, may be involved in mechanisms improving insulin sensitivity in our study population.

On the other hand, in our study we also find some genera positively associated with IR: *Faecalibacterium*, *Butyricicoccus* and *Erysipelotricaceae* UCG-003. Some *Faecalibacterium* strains, like *F. prausnitzii*, are butyrate producers used as health biomarkers in humans for its immunomodulatory properties and more specifically for its anti-inflammatory effects (52). Hippe et al., analyzed through molecular experiments based on qPCR, the different *F. prausnitzii* phylotypes and their butyrate producing capacity in non-diabetic individuals with obesity, to determine whether an excess of butyrate production can be a risk factor for the development T2D. Results shown that the overproduction of butyrate induced by different *F. prausnitzii* phylotypes discriminates between obese developing T2D or not, suggesting possible different inflammatory genesis patterns in the host (53). Other studies have suggested that the disruption in the relative proportions of butyrate-producing bacteria, such as *F. prausnitzi*, in gut microbial populations may contribute to weight gain and IR (54).

A similar argument can be extended to another genus found positively associated with HOMA-IR index, *Butyricicoccus*, which includes butyrate-producing strains considered beneficial for gut health (55). Despite the large evidence supporting the beneficial effects of butyrate on gut health, its effect on obesity is still a debated topic. Some studies shown that butyrate induces obesity providing the substrate for energy expenditure but also by engaging in signaling pathways involved in glycolipid metabolism (56). This last observation supports our findings showing butyrate-producing taxa such as *Faecalibacterium* and *Butyricicoccus* positively associated with impaired metabolism and IR.

In accordance, our findings also reveal that HOMA-IR index was positively associated with functions linked to mono-, di-, and polysaccharides degradation. Non-digestible carbohydrates are fermented by colonic microbes, leading to the production SCFAs such as butyrate (57).

We observed that taxa of interest contribute significantly to the GMM positively associated with HOMA-IR index linked to fructan degradation, glutamine degradation II, lactaldehyde degradation and lactate production. Indeed, dietary fibers are the main source of oligosaccharides such as fructan, which can promote the growth of butyrate-producing bacteria (58). In addition, lactate and lactaldehyde are fermentation products intermediary in the production of butyrate from carbohydrates (59, 60). Accordingly, it seems that the action of some taxa of interest is mediated by functions mainly linked to butyrate production, and that this metabolite is playing a role in the association observed with IR.

*Erysipelotrichaceae* have been found enriched in case of inflammatory diseases and obesity, probably because of its involvement in host lipid metabolism (61). In addition, it was found associated with impaired glucose metabolism (62). Accordingly, we observed a positive association between this genus and high levels of IR. Bacteria belonging to *Erysipelotrichaceae* family use proteins and saccharides as main substrates for lactate production, one of the functional metabolic pathways involved in our study (60).

Unsurprisingly, we discovered that the taxa associated with IR were inferred to contribute to several of the GMM also associated with IR. However, we also found that the degree to which these taxa contributed to the respective functions was dependent on IR as well. This may have major implications for our understanding of the role of the microbiome in IR and indeed other conditions altering host gut metabolism. The altered gut metabolic environment promotes the relative abundance of different microbes that perform the same functions, in a manner similar to that described by Zhang and colleagues in their work on ecological guilds (63).

However, both in the taxonomic and functional aspect, previous studies shown discrepancies, perhaps partially explained by the inherent biases in metagenomic sequencing which make it problematic to estimate bacterial abundance from sequencing data (64). It is also important to mention that to overpass such bias we used a compositional approach for the microbiome analysis (65). Furthermore, is also worth to mention that the sequencing approach conducted in this study is also prone to bias, as described by Barb and colleagues (66). More complete and complex studies, including deepen microbial community profiling together with biochemical characterization of the environment, are needed in order to better understand the taxonomic and functional signatures related to IR.

There were several limitations in this study. First, due to the nature of 16S sequencing, we were limited to a genus-level resolution. Similarly, though PICRUSt2 has been shown to be reasonably reliable, especially in humans, we were restricted by the number and quality of the genomes present in the database used to infer the functional potential of the microbiome. For the same reasons, we were unable to assess the non-bacterial fraction of the microbiome. Whole genome shotgun metagenomics addresses both of these limitations, as it enables the detection and relative quantification of non-bacterial microbes as well as the bacteriome, and also enables analysis at a higher resolution and should be considered in the future. Another important point to consider is the large inherent variance in the microbiome, which is often confounded by host variables (67). The PREDIMED-Plus cohort by its nature consists late-middle aged and overweight participants. It is likely that some of the findings cannot be generalized to all ages and weights. Furthermore, this study only had a single microbiome measurement per participant, thus rendering stability and volatility analysis of the microbiome beyond our reach (68, 69). We eagerly await future studies and subsequent meta-analyses that will shed further light on this. Furthermore, given the observational nature of this study, it was not possible to conclude on causality or directionality. Future experiments using the fecal microbiota transplantation (FMT) technique could be considered to elucidate this last aspect.

## Conclusions

In the current study we were able to identify taxa and functions associated with IR and to suggest that bacteria contribute differentially to these functions, also depending on IR. We observed genera *Desulfovibrio*, *Odoribacter* and *Oscillospiraceae* UCG-002 negatively associated with IR through mechanism of amino acid degradation that involves H_2_S, activation of gluconeogenesis, immunomodulation and beneficial effects of acetate. On the other hand, we observed that the positive association with IR involves genera such as *Feacalibaterium* and *Butyricicoccus* and GMM linked with the production of butyrate. To conclude, these findings are promising especially for the perspective of tailoring therapeutic strategies based on the identification of specific signatures of the gut bacterial community and using this information to improve the treatments for IR and other metabolic impairments related to obesity. However, some further investigation, especially under the metabolic point of view, are needed in order to strengthen these findings.

## Data Availability Statement

The datasets generated and analyzed during the current study are not publicly available due to data regulations and for ethical reasons, considering that this information might compromise research participants’ consent because our participants only gave their consent for the use of their data by the original team of investigators. However, collaboration for data analyses can be requested by sending a letter to the PREDIMED-Plus steering Committee (predimed_plus_scommittee@googlegroups.com). The request will then be passed to all the members of the PREDIMED-Plus Steering Committee for deliberation.

## Ethics Statement

The study was conducted according to the guidelines of the Declaration of Helsinki, and approved by the Hospital Universitari Sant Joan de Reus Ethics Committee (protocol code 13-07-25/7proj2, date of approval: 25/07/2013), and the Comité de Ética de la Investigación Provincial de Málaga (protocol code Predimed+DM/01, date of approval: 27/11/2014). The patients/participants provided their written informed consent to participate in this study.

## Author Contributions

Conceptualization, AA, FJT, JeV, DC, JoV, MF, MB, and JSS. Formal analysis, AA and TFSB. Funding acquisition, FJT, JeV, DC, JoV, MF, and JSS. Writing—original draft preparation, AA. Writing—review and editing, TFSB, MB, and JSS. Supervision, MB and JSS. All authors have read and agreed to the published version of the manuscript.

## Funding

This research was funded by the European Union’s Horizon 2020 research and innovation programme under the Marie Skłodowska-Curie grant agreement No. 713679 and from the Universitat Rovira i Virgili (URV). This work was supported by the official Spanish Institutions for funding scientific biomedical research, CIBER Fisiopatología de la Obesidad y Nutrición (CIBEROBN) and Instituto de Salud Carlos III (ISCIII), through the Fondo de Investigación para la Salud (FIS), which is co-funded by the European Regional Development Fund (three coordinated FIS projects led by JJS, including the following projects: PI13/00462, PI16/00501 and PI19/00576); the Especial Action Project entitled: Implementación y evaluación de una intervención intensiva sobre la actividad física Cohorte PREDIMED-Plus grant to JSS; the Recercaixa (number 2013ACUP00194) grant to JSS; The Fondo de Investigaciones Sanitarias of the Instituto de Salut Carlos III PI17/00215; The Generalitat Valenciana PROMETEO 17/2017 and PROMETEO 21/2021. None of the funding sources took part in the design, collection, analysis, interpretation of the data, or writing the report, or in the decision to submit the manuscript for publication. JSS, senior author of this study gratefully acknowledges the financial support by ICREA under the ICREA Academia programme. Food companies Hojiblanca (Lucena, Spain) and Patrimonio Comunal Olivarero (Madrid, Spain) donated extra virgin olive oil; and the Almond Board of California (Modesto, CA), American Pistachio Growers (Fresno, CA), and Paramount Farms (Wonderful Company, LLC, Los Angeles, CA) donated nuts for the PREDIMED- Pilot.

## Conflict of Interest

The authors declare that the research was conducted in the absence of any commercial or financial relationships that could be construed as a potential conflict of interest.

## Publisher’s Note

All claims expressed in this article are solely those of the authors and do not necessarily represent those of their affiliated organizations, or those of the publisher, the editors and the reviewers. Any product that may be evaluated in this article, or claim that may be made by its manufacturer, is not guaranteed or endorsed by the publisher.

## References

[1] Gurung M Li Z You H Rodrigues R Jump DB Morgun A. Role of Gut Microbiota in Type 2 Diabetes Pathophysiology. EBioMedicine (2020) 51:102590. doi: 10.1016/j.ebiom.2019.11.051 31901868 PMC6948163

[2] Martyn JAJ Kaneki M Yasuhara S Warner DS Warner MA. Obesity-Induced Insulin Resistance and Hyperglycemia. Anesthesiology (2008) 109:137–48. doi: 10.1097/ALN.0b013e3181799d45 PMC3896971 18580184

[3] Fahed M Abou Jaoudeh MG Merhi S Mosleh JMB Ghadieh R Ghadieh R. Evaluation of Risk Factors for Insulin Resistance: A Cross Sectional Study Among Employees at a Private University in Lebanon. BMC Endocr Disord (2020) 20:1–14. doi: 10.1186/s12902-020-00558-9 32522257 PMC7288486

[4] Saad MJA Santos A Prada PO. Linking Gut Microbiota and Inflammation to Obesity and Insulin Resistance. Physiology (2016) 31:283–93. doi: 10.1152/physiol.00041.2015 27252163

[5] Bäckhed F Ding H Wang T Hooper LV Gou YK Nagy A. The Gut Microbiota as an Environmental Factor That Regulates Fat Storage. Proc Natl Acad Sci USA. (2004) 101:15718–23. doi: 10.1073/pnas.0407076101 PMC524219 15505215

[6] Vrieze A Van Nood E Holleman F Salojärvi J Kootte RS Bartelsman JFWM. Transfer of Intestinal Microbiota From Lean Donors Increases Insulin Sensitivity in Individuals With Metabolic Syndrome. Gastroenterology (2012) 143:913–6.e7 doi: 10.1053/j.gastro.2012.06.031 22728514

[7] Cani PD Amar J Iglesias MA Poggi M Knauf C Bastelica D. Metabolic Endotoxemia Initiates Obesity and Insulin Resistance. Diabetes (2007) 57:1761–72 doi: 10.2337/db06-1491 17456850

[8] Amar J Chabo C Waget A Klopp P Vachoux C Bermúdez-Humarán LG. Intestinal Mucosal Adherence and Translocation of Commensal Bacteria at the Early Onset of Type 2 Diabetes: Molecular Mechanisms and Probiotic Treatment. EMBO Mol Med (2011) 3:559–72. doi: 10.1002/emmm.201100159 PMC3265717 21735552

[9] Caricilli AM Picardi PK de Abreu LL Ueno M Prada PO Ropelle ER. Gut Microbiota is a Key Modulator of Insulin Resistance in TLR 2 Knockout Mice. PloS Biol (2011) 14:e1002479. doi: 10.1371/journal.pbio.1001212 PMC4876994 27213533

[10] Medzhitov R Horng T. Transcriptional Control of the Inflammatory Response. Nat Rev Immunol (2009) 9:692–703. doi: 10.1038/nri2634 19859064

[11] Tan J McKenzie C Potamitis M Thorburn AN Mackay CR Macia L. The Role of Short-Chain Fatty Acids in Health and Disease. Adv Immunol (2014) 91–119. doi: 10.1016/B978-0-12-800100-4.00003-9. 24388214

[12] Li M van Esch BCAM Wagenaar GTM Garssen J Folkerts G Henricks PAJ. Pro- and Anti-Inflammatory Effects of Short Chain Fatty Acids on Immune and Endothelial Cells. Eur J Pharmacol (2018) 831:52–9. doi: 10.1016/j.ejphar.2018.05.003 29750914

[13] Ratajczak W Rył A Mizerski A Walczakiewicz K Sipak O Laszczyńska M. Immunomodulatory Potential of Gut Microbiome-Derived Short-Chain Fatty Acids (SCFAs). Acta Biochim Pol (2019) 66:1–12. doi: 10.18388/abp.2018_2648 30831575

[14] Perry RJ Peng L Barry NA Cline GW Zhang D Cardone RL. Acetate Mediates a Microbiome-Brain-β-Cell Axis to Promote Metabolic Syndrome. Nature (2016) 534:213–7. doi: 10.1038/nature18309 PMC4922538 27279214

[15] Wahlström A Sayin SI Marschall HU Bäckhed F. Intestinal Crosstalk Between Bile Acids and Microbiota and Its Impact on Host Metabolism. Cell Metab (2016) 24:41–50. doi: 10.1016/j.cmet.2016.05.005 27320064

[16] Gérard C Vidal H. Impact of Gut Microbiota on Host Glycemic Control. Front Endocrinol (Lausanne) (2019) 10:29. doi: 10.3389/fendo.2019.00029 30761090 PMC6363653

[17] Pedersen HK Gudmundsdottir V Nielsen HB Hyotylainen T Nielsen T Jensen BAH. Human Gut Microbes Impact Host Serum Metabolome and Insulin Sensitivity. Nature (2016) 535:376–81. doi: 10.1038/nature18646 27409811

[18] Salas-Salvadó J Díaz-López A Ruiz-Canela M Basora J Fitó M Corella D. Effect of a Lifestyle Intervention Program With Energy-Restricted Mediterranean Diet and Exercise on Weight Loss and Cardiovascular Risk Factors: One-Year Results of the PREDIMED-Plus Trial. Diabetes Care (2019) 44:777–88 doi: 10.2337/dc18-0836 30389673

[19] Sayón-Orea C Razquin C Bulló M Corella D Fitó M Romaguera D. Effect of a Nutritional and Behavioral Intervention on Energy-Reduced Mediterranean Diet Adherence Among Patients With Metabolic Syndrome: Interim Analysis of the PREDIMED-Plus Randomized Clinical Trial. JAMA - J Am Med Assoc (2019) 322:1486–99. doi: 10.1001/jama.2019.14630 PMC6802271 31613346

[20] Matthews DR Hosker JP Rudenski AS Naylor BA Treacher DF Turner RC. Homeostasis Model Assessment: Insulin Resistance and β-Cell Function From Fasting Plasma Glucose and Insulin Concentrations in Man. Diabetologia (1985) 28:412–9. doi: 10.1007/BF00280883 3899825

[21] Schröder H Zomeño MD Martínez-González MA Salas-Salvadó J Corella D Vioque J. Validity of the Energy-Restricted Mediterranean Diet Adherence Screener. Clin Nutr (2021) 40:4971–9. doi: 10.1016/j.clnu.2021.06.030 34364236

[22] MacPherson CW Mathieu O Tremblay J Champagne J Nantel A Girard SA. Gut Bacterial Microbiota and its Resistome Rapidly Recover to Basal State Levels After Short-Term Amoxicillin-Clavulanic Acid Treatment in Healthy Adults. Sci Rep (2018) 8:1–14. doi: 10.1038/s41598-018-29229-5 30046129 PMC6060159

[23] Callahan BJ McMurdie PJ Rosen MJ Han AW Johnson AJA Holmes SP. DADA2: High-Resolution Sample Inference From Illumina Amplicon Data. Nat Methods (2016) 13:581–3. doi: 10.1038/nmeth.3869 PMC4927377 27214047

[24] Quast C Pruesse E Yilmaz P Gerken J Schweer T Yarza P. The SILVA Ribosomal RNA Gene Database Project: Improved Data Processing and Web-Based Tools. Nucleic Acids Res (2013) 41:D590–6. doi: 10.1093/nar/gks1219 PMC3531112 23193283

[25] Aitchison J Barceló-Vidal C Martín-Fernández JA Pawlowsky-Glahn V. Logratio Analysis and Compositional Distance. Math Geol (2000) 32:271–5. doi: 10.1023/A:1007529726302

[26] Chao A. Estimating the Population Size for Capture-Recapture Data With Unequal Catchability. Biometrics (1987) 43:783. doi: 10.2307/2531532 3427163

[27] Shannon CE. A Mathematical Theory of Communication. Bell Syst Tech J (1948) 27:623–56. doi: 10.1002/j.1538-7305.1948.tb00917.x

[28] Simpson EH. Measurment of Diversity. Nature (1949) 163:688. doi: 10.1038/163688a0

[29] Storey JD Tibshirani R. Statistical Significance for Genomewide Studies. Proc Natl Acad Sci (2003) 100:9440–5. doi: 10.1073/pnas.1530509100 PMC170937 12883005

[30] Douglas GM Maffei VJ Zaneveld JR Yurgel SN Brown JR Taylor CM. PICRUSt2 for Prediction of Metagenome Functions. Nat Biotechnol (2020) 38:685–8. doi: 10.1038/s41587-020-0548-6 PMC7365738 32483366

[31] Vieira-Silva S Falony G Darzi Y Lima-Mendez G Garcia Yunta R Okuda S. Species-Function Relationships Shape Ecological Properties of the Human Gut Microbiome. Nat Microbiol (2016) 1:1–8. doi: 10.1038/nmicrobiol.2016.88 27573110

[32] The Human Microbiome Project Consortium. Structure, Function and Diversity of the Healthy Human Microbiome. Nature (2012) 486:207–14. doi: 10.1038/nature11234 PMC3564958 22699609

[33] Kushkevych I Dordević D Kollár P. Analysis of Physiological Parameters of Desulfovibrio Strains From Individuals With Colitis. Open Life Sci (2018) 13:481–8. doi: 10.1515/biol-2018-0057 PMC7874683 33817117

[34] Rowan F Docherty NG Murphy M Murphy B Coffey JC O’Connell PR. Desulfovibrio Bacterial Species Are Increased in Ulcerative Colitis. Dis Colon Rectum (2010) 53:1530–6. doi: 10.1007/DCR.0b013e3181f1e620 20940602

[35] Zhang C Zhang M Wang S Han R Cao Y Hua W. Interactions Between Gut Microbiota, Host Genetics and Diet Relevant to Development of Metabolic Syndromes in Mice. ISME J (2010) 4:232–41. doi: 10.1038/ismej.2009.112 19865183

[36] Andoh A Nishida A Takahashi K Inatomi O Imaeda H Bamba S. Comparison of the Gut Microbial Community Between Obese and Lean Peoples Using 16S Gene Sequencing in a Japanese Population. J Clin Biochem Nutr (2016) 59:65–70. doi: 10.3164/jcbn.15-152 27499582 PMC4933688

[37] Karlsson CLJ Önnerfält J Xu J Molin G Ahrné S Thorngren-Jerneck K. The Microbiota of the Gut in Preschool Children With Normal and Excessive Body Weight. Obesity (2012) 20:2257–61. doi: 10.1038/oby.2012.110 22546742

[38] Garcia-Mantrana I Selma-Royo M Alcantara C Collado MC. Shifts on Gut Microbiota Associated to Mediterranean Diet Adherence and Specific Dietary Intakes on General Adult Population. Front Microbiol (2018) 9:890. doi: 10.3389/fmicb.2018.00890 29867803 PMC5949328

[39] Pichette J Fynn-Sackey N Gagnon J. Hydrogen Sulfide and Sulfate Prebiotic Stimulates the Secretion of GLP-1 and Improves Glycemia in Male Mice. Endocrinology (2017) 158:3416–25. doi: 10.1210/en.2017-00391 28977605

[40] Wang L Li P Tang Z Yan X Feng B. Structural Modulation of the Gut Microbiota and the Relationship With Body Weight: Compared Evaluation of Liraglutide and Saxagliptin Treatment. Sci Rep (2016) 6:33251. doi: 10.1038/srep33251 27633081 PMC5025740

[41] Neis EPJG Dejong CHC Rensen SS. The Role of Microbial Amino Acid Metabolism in Host Metabolism. Nutrients (2015) 7:2930–46 doi: 10.3390/nu7042930 PMC4425181 25894657

[42] Sircana A Framarin L Leone N Berrutti M Castellino F Parente R. Altered Gut Microbiota in Type 2 Diabetes: Just a Coincidence? Curr Diabetes Rep (2018) 18:98. doi: 10.1007/s11892-018-1057-6 30215149

[43] Orsso CE Peng Y Deehan EC Tan Q Field CJ Madsen KL. Composition and Functions of the Gut Microbiome in Pediatric Obesity: Relationships With Markers of Insulin Resistance. Microorganisms (2021) 9:1–18. doi: 10.3390/microorganisms9071490 PMC8304481 34361925

[44] Zhao J Zhang X Liu H Brown MA Qiao S. Dietary Protein and Gut Microbiota Composition and Function. Curr Protein Pept Sci (2018) 20:145–54. doi: 10.2174/1389203719666180514145437 29756574

[45] Hernández MAG Canfora EE Jocken JWE Blaak EE. The Short-Chain Fatty Acid Acetate in Body Weight Control and Insulin Sensitivity. Nutrients (2019) 11:1943. doi: 10.3390/nu11081943 PMC6723943 31426593

[46] Gomez-Arango LF Barrett HL McIntyre HD Callaway LK Morrison M Dekker Nitert M. Increased Systolic and Diastolic Blood Pressure is Associated With Altered Gut Microbiota Composition and Butyrate Production in Early Pregnancy. Hypertension (2016) 68:974–81. doi: 10.1161/HYPERTENSIONAHA.116.07910 27528065

[47] Serena C Ceperuelo-Mallafré V Keiran N Queipo-Ortuño MI Bernal R Gomez-Huelgas R. Elevated Circulating Levels of Succinate in Human Obesity are Linked to Specific Gut Microbiota. ISME J (2018) 12:1642–57. doi: 10.1038/s41396-018-0068-2 PMC6018807 29434314

[48] Brahe LK Le Chatelier E Prifti E Pons N Kennedy S Hansen T. Specific Gut Microbiota Features and Metabolic Markers in Postmenopausal Women With Obesity. Nutr Diabetes (2015) 5:e159–7. doi: 10.1038/nutd.2015.9 PMC4491860 26075636

[49] Wang Y Tang C Tang Y Yin H Liu X. Capsaicin has an Anti-Obesity Effect Through Alterations in Gut Microbiota Populations and Short-Chain Fatty Acid Concentrations. Food Nutr Res (2020) 64:1–14. doi: 10.29219/fnr.v64.3525 PMC7054644 32180694

[50] Biassoni R Di Marco E Squillario M Barla A Piccolo G Ugolotti E. Gut Microbiota in T1DM-Onset Pediatric Patients: Machine-Learning Algorithms to Classify Microorganisms as Disease Linked. J Clin Endocrinol Metab (2020) 105:e3114–26. doi: 10.1210/clinem/dgaa407 32692360

[51] Maya-Lucas O Murugesan S Nirmalkar K Alcaraz LD Hoyo-Vadillo C Pizano-Zárate ML. The Gut Microbiome of Mexican Children Affected by Obesity. Anaerobe (2019) 55:11–23. doi: 10.1016/j.anaerobe.2018.10.009 30366118

[52] Lenoir M Martín R Torres-Maravilla E Chadi S González-Dávila P Sokol H. Butyrate Mediates Anti-Inflammatory Effects of Faecalibacterium Prausnitzii in Intestinal Epithelial Cells Through Dact3. Gut Microbes (2020) 12:1–16. doi: 10.1080/19490976.2020.1826748 PMC7567499 33054518

[53] Hippe B Remely M Aumueller E Pointner A Magnet U Haslberger AG. Faecalibacterium Prausnitzii Phylotypes in Type Two Diabetic, Obese, and Lean Control Subjects. Benef Microbes (2016) 7:511–7. doi: 10.3920/BM2015.0075 27048834

[54] Barlow GM Yu A Mathur R. Role of the Gut Microbiome in Obesity and Diabetes Mellitus. Nutr Clin Pract (2015) 30:787–97. doi: 10.1177/0884533615609896 26452391

[55] Ma Q Li Y Wang J Li P Duan Y Dai H. Investigation of Gut Microbiome Changes in Type 1 Diabetic Mellitus Rats Based on High-Throughput Sequencing. BioMed Pharmacother (2020) 124:109873. doi: 10.1016/j.biopha.2020.109873 31986412

[56] Liu H Wang J He T Becker S Zhang G Li D. Butyrate: A Double-Edged Sword for Health? Adv Nutr (2018) 9:21–9. doi: 10.1093/advances/nmx009 PMC6333934 29438462

[57] Blandino G Inturri R Lazzara F Di Rosa M Malaguarnera L. Impact of Gut Microbiota on Diabetes Mellitus. Diabetes Metab (2016) 42:303–15. doi: 10.1016/j.diabet.2016.04.004 27179626

[58] Bibbò S Ianiro G Giorgio V Scaldaferri F Masucci L Gasbarrini A. The Role of Diet on Gut Microbiota Composition. Eur Rev Med Pharmacol Sci (2016) 20:4742–9. 27906427

[59] Louis P Flint HJ. Formation of Propionate and Butyrate by the Human Colonic Microbiota. Environ Microbiol (2017) 19:29–41. doi: 10.1111/1462-2920.13589 27928878

[60] Oliphant K Allen-Vercoe E. Macronutrient Metabolism by the Human Gut Microbiome: Major Fermentation by-Products and Their Impact on Host Health. Microbiome (2019) 7:1–15. doi: 10.1186/s40168-019-0704-8 31196177 PMC6567490

[61] Kaakoush NO. Insights Into the Role of Erysipelotrichaceae in the Human Host. Front Cell Infect Microbiol (2015) 5:84. doi: 10.3389/fcimb.2015.00084 26636046 PMC4653637

[62] Lippert K Kedenko L Antonielli L Kedenko I Gemeier C Leitner M. Gut Microbiota Dysbiosis Associated With Glucose Metabolism Disorders and the Metabolic Syndrome in Older Adults. Benef Microbes (2017) 8:545–56. doi: 10.3920/BM2016.0184 28701081

[63] Zhang C Yin A Li H Wang R Wu G Shen J. Dietary Modulation of Gut Microbiota Contributes to Alleviation of Both Genetic and Simple Obesity in Children. EBioMedicine (2015) 2:968–84. doi: 10.1016/j.ebiom.2015.07.007 PMC4563136 26425705

[64] McLaren MR Willis AD Callahan BJ. Consistent and Correctable Bias in Metagenomic Sequencing Experiments. Elife (2019) 8:1–31. doi: 10.7554/eLife.46923 PMC6739870 31502536

[65] Gloor GB Macklaim JM Pawlowsky-Glahn V Egozcue JJ. Microbiome Datasets are Compositional: And This is Not Optional. Front Microbiol (2017) 8:2224. doi: 10.3389/fmicb.2017.02224 29187837 PMC5695134

[66] Barb JJ Oler AJ Kim H-S Chalmers N Wallen GR Cashion A. Development of an Analysis Pipeline Characterizing Multiple Hypervariable Regions of 16S rRNA Using Mock Samples. PloS One (2016) 11:e0148047. doi: 10.1371/journal.pone.0148047 26829716 PMC4734828

[67] Vujkovic-Cvijin I Sklar J Jiang L Natarajan L Knight R Belkaid Y. Host Variables Confound Gut Microbiota Studies of Human Disease. Nature (2020) 587:448–54. doi: 10.1038/s41586-020-2881-9 PMC7677204 33149306

[68] Vandeputte D De Commer L Tito RY Kathagen G Sabino J Vermeire S. Temporal Variability in Quantitative Human Gut Microbiome Profiles and Implications for Clinical Research. Nat Commun (2021) 12:6740. doi: 10.1038/s41467-021-27098-7 34795283 PMC8602282

[69] Bastiaanssen TF Gururajan A van de Wouw M Moloney GM Ritz NL Long-Smith CM. Volatility as a Concept to Understand the Impact of Stress on the Microbiome. Psychoneuroendocrinology (2021) 124:105047. doi: 10.1016/j.psyneuen.2020.105047 33307493

