# Supplementary material for: Taxonomic and Functional Fecal Microbiota Signatures Associated With Insulin Resistance in Non-Diabetic Subjects With Overweight/Obesity Within the Frame of the PREDIMED-Plus Study
Source: Front Endocrinol (Lausanne). 2022 Apr 28;13:804455. doi: 10.3389/fendo.2022.804455 (PMC9097279; doi:10.3389/fendo.2022.804455)

SUPPLEMENTAL MATERIAL

Figure S1 | Principal component analysis plot.

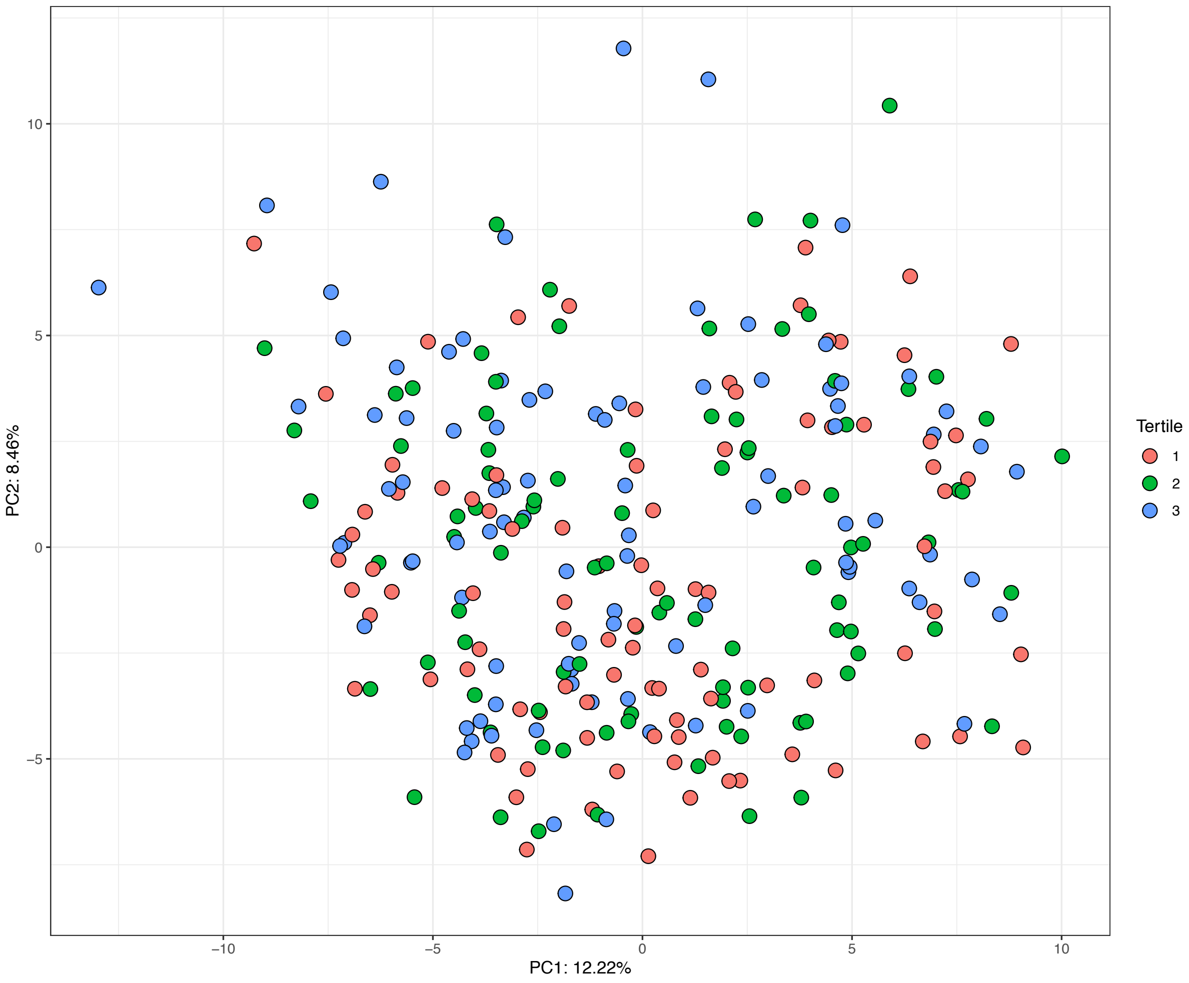

Figure S2 | Box plot showing differences in Chao1, Shannon and Simpson indices between tertiles of HOMA-IR index.

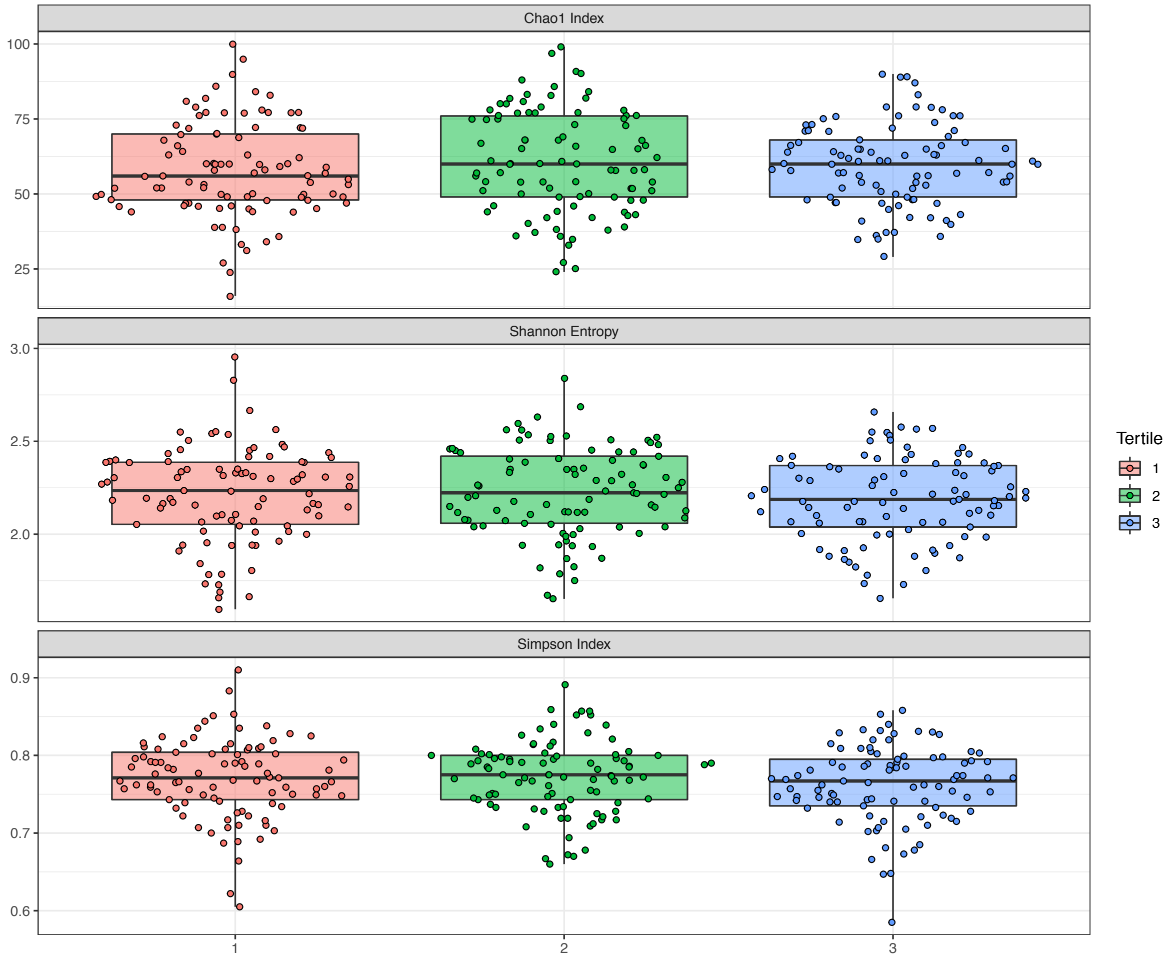

Table S1 | Differences in alpha diversity indices Chao1, Simpson, Shannon, between tertiles of HOMA-IR index.

	**Tertile 1**	**Tertile 2**	**Tertile 3**	***p*-value**
Chao1	60.0 ± 16.2	57.2 ± 15.1	57.9 ± 15.5	0.446
Simpson	0.8 [0.1]	0.8 [0.1]	0.9 [0.1]	0.720
Shannon	2.6 ± 0.4	2.6 ± 0.4	2.6 ± 0.4	0.855

Data presented as mean ± SD or median [IQR]. One-way ANOVA test and Kruskal-Wallis test used to calculate differences across tertiles of HOMA-IR; $p < 0.05$ deemed as significant.

Table S2 | Results of PERMANOVA analysis and permutation test for homogeneity of multivariate dispersions using Aitchison distance.

	**Df**	**Sums of squares**	**Mean squares**	**F.Model**	**R2**	**Pr(>F)**
Tertiles	2	281	140.26	0.8247	0.00589	0.802198
Recruiting c.	1	545	544.59	3.2022	0.01144	0.000999 ***
Residuals	274	46769	170.07		0.98266	
Total	278	47594			1.00000	

Figure S3 | Taxa contribution (per sample) to the GMMs negatively associated with HOMA-IR index. Bacteria that did not contribute more than 30% to any function were classified as "other taxa".

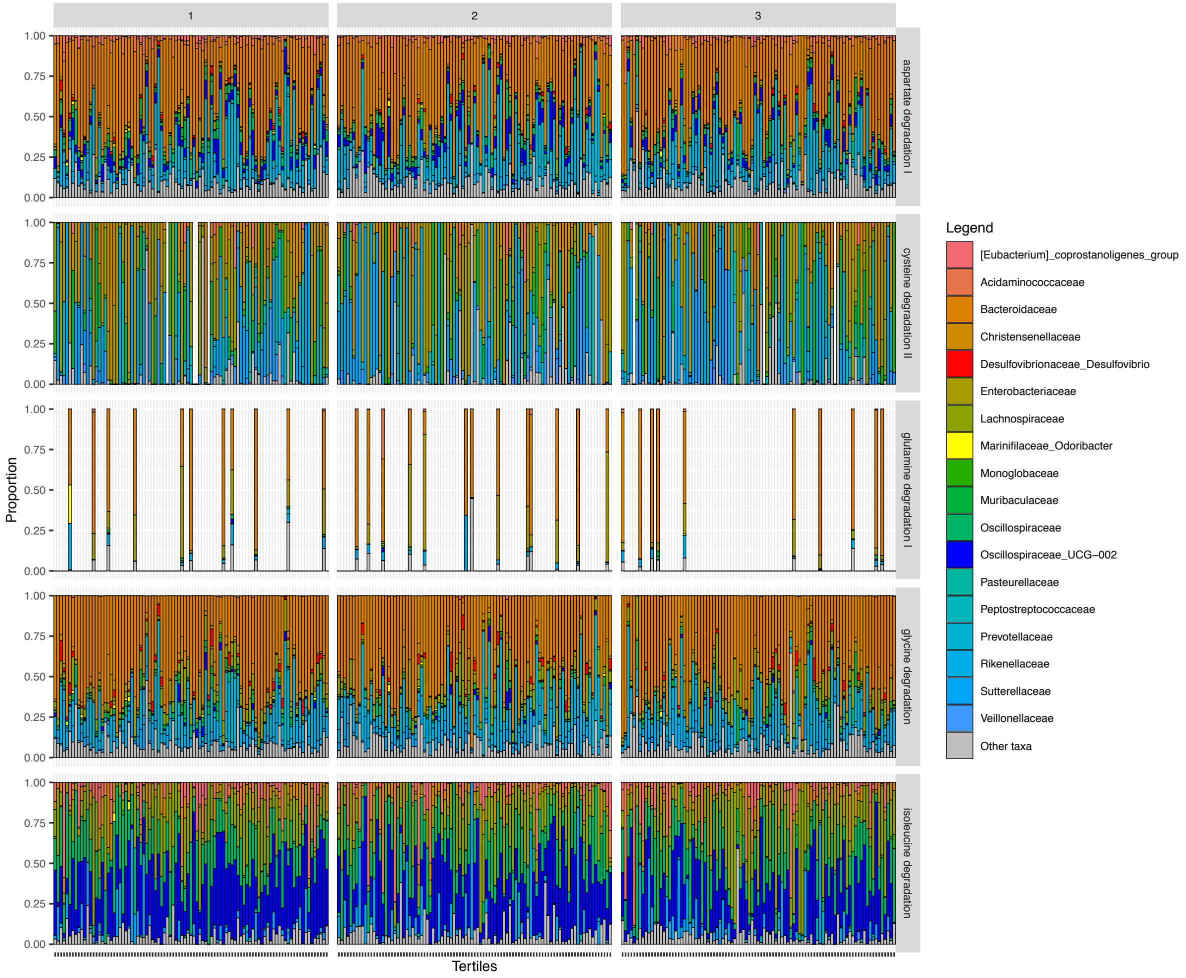

Figure S4 | Taxa contribution (per sample) to the GMMs positively associated with HOMA-IR index. Bacteria that did not contribute more than 30% to any function were classified as "other taxa".

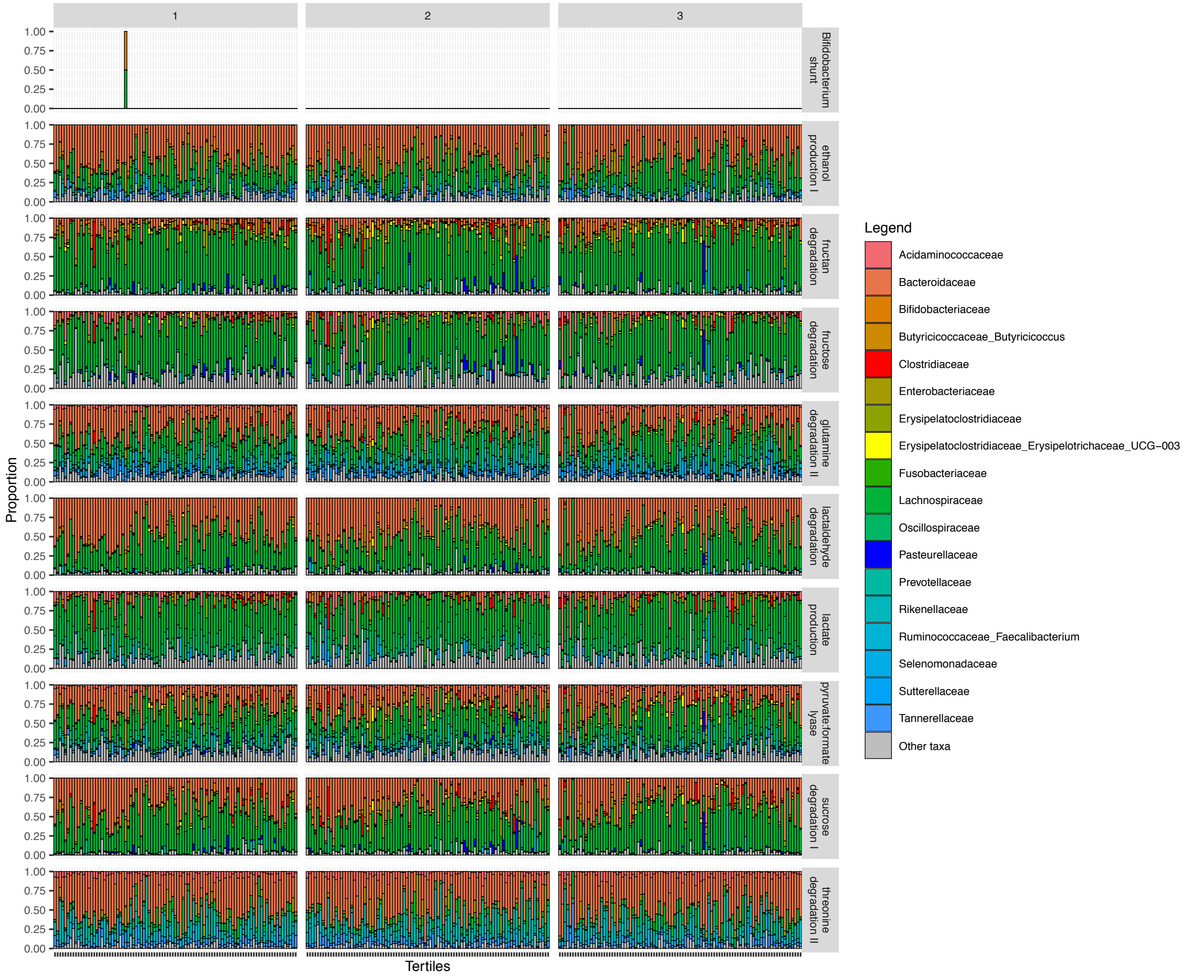

Figure S5 | Taxa contribution (per tertile) to the GMMs negatively associated with HOMA-IR index. Bacteria that did not contribute more than 30% to any function were classified as "other taxa".

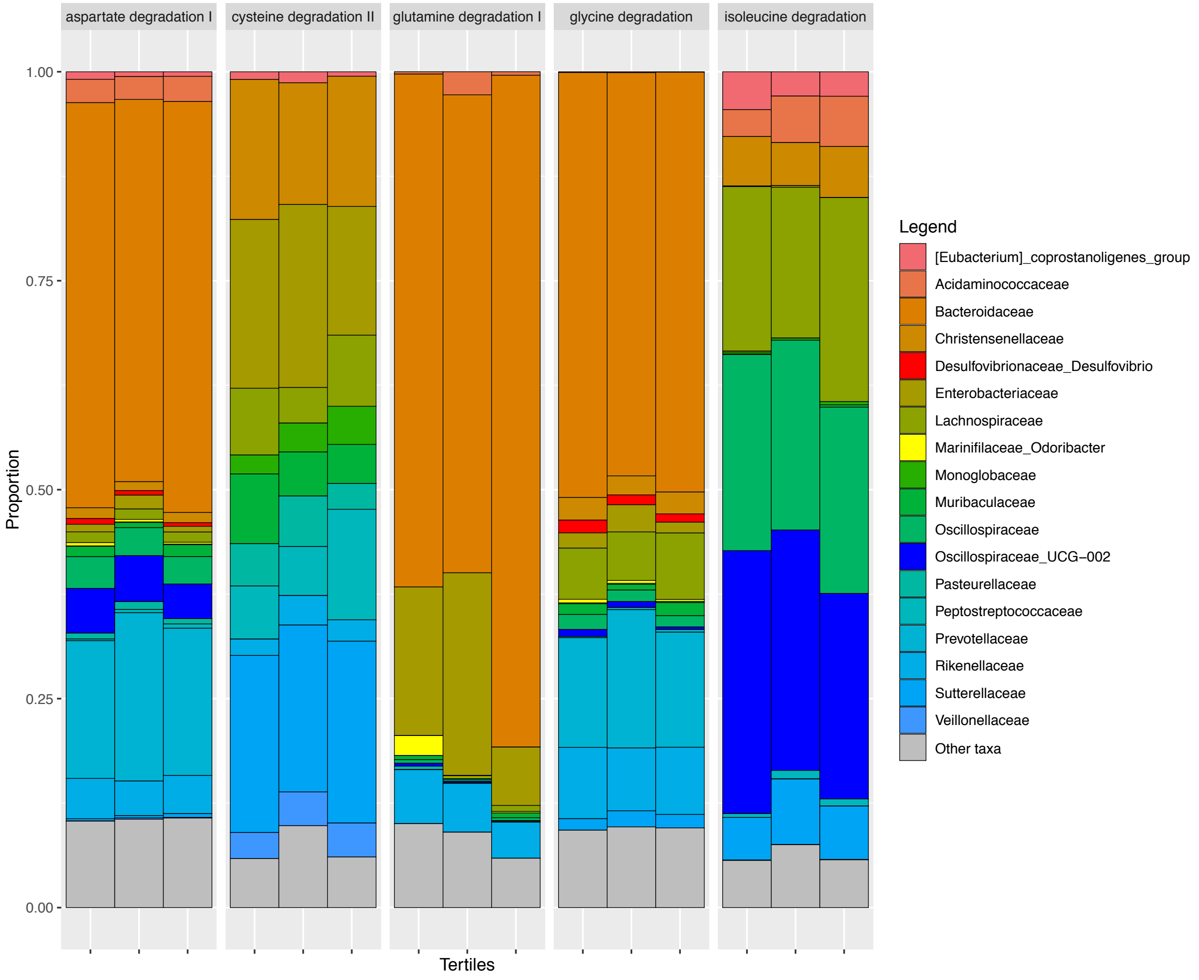

Figure S6 | Taxa contribution (per tertile) to the GMMs positively associated with HOMA-IR index. Bacteria that did not contribute more than 30% to any function were classified as "other taxa".

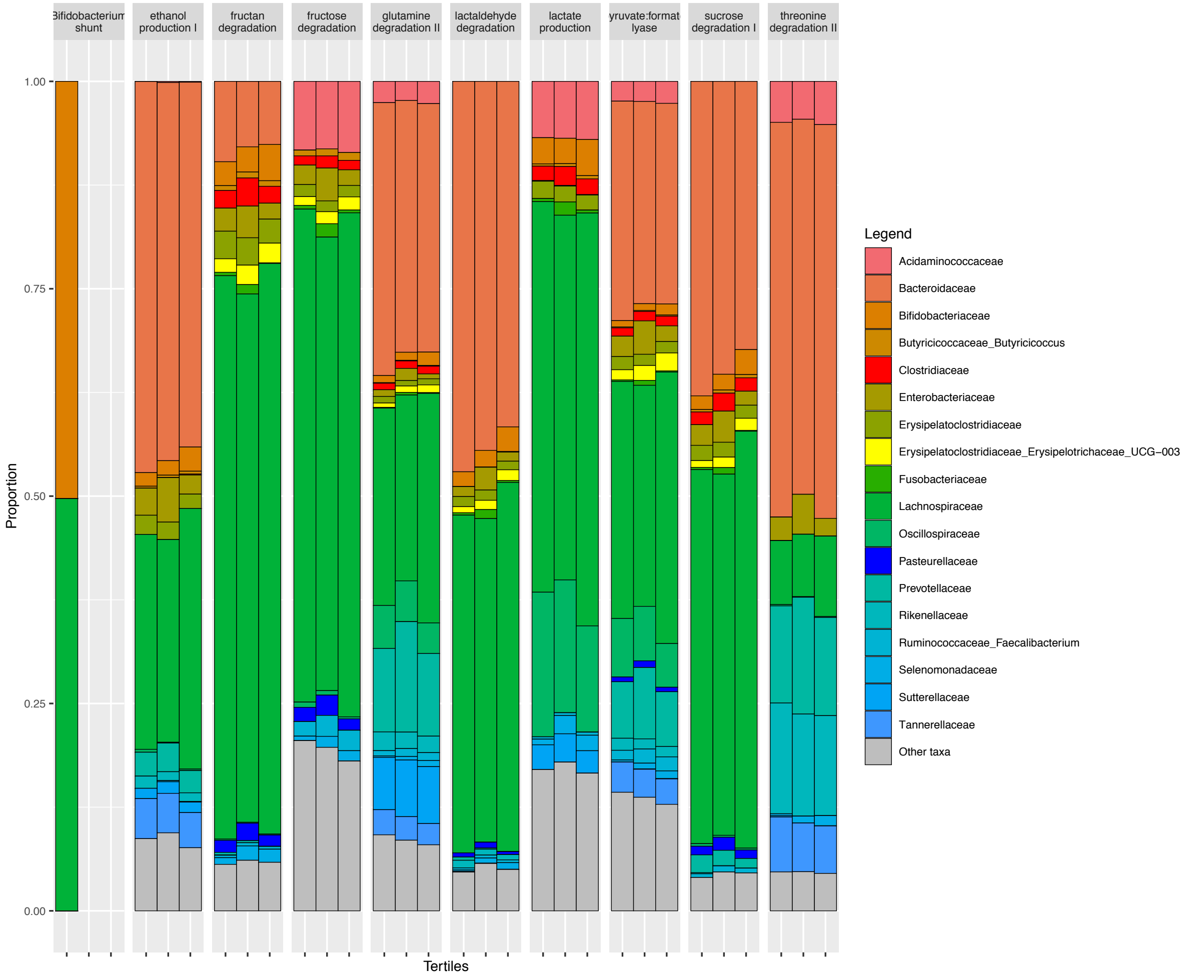

Supplement: Supplementary file 1 [file DataSheet_1.pdf]
